# SAR Image Formation Method with Azimuth Periodically Missing Data Based on RELAX Algorithm

**DOI:** 10.3390/s21010049

**Published:** 2020-12-24

**Authors:** Weixing Yang, Daiyin Zhu

**Affiliations:** Key Laboratory of Radar Imaging and Microwave Photonics & Ministry of Education, College of Electronic and Information Engineering, Nanjing University of Aeronautics and Astronautics, Nanjing 210016, China; weixing_nuaa@nuaa.edu.cn

**Keywords:** synthetic aperture radar, azimuth periodically missing data, RELAX algorithm

## Abstract

Synthetic aperture radar (SAR) is a widely used remote sensing observation technique. However, SAR raw echo data may be lost during the process of data acquisition by radar platform. In this paper, the imaging problem of SAR echo signal with periodically missing data along the azimuth is analyzed and a novel imaging method is proposed. Firstly, the problem of artificial artifact targets caused by periodically missing data is explained in detail, and the corresponding mathematical model is established. Then, the recovery method based on the RELAX algorithm with periodic notches data is proposed. In addition, when the size of two-dimensional (2D) echo data are large, block restoration along the azimuth is proposed to reduce the amount of calculation. Finally, the advantages of the algorithm proposed in this paper is demonstrated by the points target simulated SAR echo data processing and the real raw SAR echo data processing. When the azimuth periodically missing data rate is 50%, the SAR echo data can be recovered and the well-focused image can be obtained. Comparing the image entropy value and structural similarity index (SSIM) of the focused image, it proves the superiority of the proposed algorithm in solving the imaging problem of SAR azimuth periodically missing data.

## 1. Introduction

Synthetic Aperture Radar (SAR) is an important technology in modern remote sensing observation field. The radar system can realize high-resolution two-dimensional (2D) images of regions of interest [1]. With the characteristics of all-day, all-weather, and high-resolution imaging, SAR has many applications in military reconnaissance, civil monitoring, and other fields. In the SAR imaging system, the high resolution in azimuth is realized by using the radar platform to fly for a period, and the motion of “small antenna” is equivalent to synthesizing a “large antenna” [2]. However, interference during the flight of the radar platform and damage to the recorded data will result in missing data along the azimuth [3]. In modern radar imaging system, there will also be data missing. Modern radar systems generally integrate multiple functions and they can simultaneously search, track, and complete automatic targets classification. Switching between multiple operating modes will result in incomplete SAR phase history data [4,5,6]. The azimuth missing data will seriously deteriorate the imaging quality, cause the appearance of artificial artifact targets and affect the post-processing such as target classification and image recognition and so on. Therefore, solving the problem of SAR imaging with azimuth missing data is an important research content in the field of SAR imaging.

According to the shape of missing data, there are two patterns of missing data along the azimuth, one is the random gaps missing data, and the other is the periodic gaps missing data [7]. The randomly missing data is more common in SAR systems. The periodic continuous missing data is a special case, which will occur in the continuous interrupted SAR imaging system. In spaceborne or airborne SAR imaging system, interrupted SAR operation mode is proposed, which uses single antenna to transmit and receive signals. This operation mode can realize the low cost, miniaturization and lightweight of SAR system, and has important research prospects. When the SAR system adopts the interrupted mode, there will be periodic missing data in the azimuth. If the traditional SAR imaging algorithm is used, the azimuth periodically missing data will lead to the appearance of artifact targets along the SAR azimuth, which will deteriorate the imaging quality. SAR image formation with azimuth periodically missing data is also a difficult problem studied in recent years. Therefore, the imaging problem of SAR azimuth periodic continuous missing data is mainly studied in this paper.

For the problem of SAR imaging with periodically missing data along the azimuth, a large number of methods have been proposed to eliminate azimuth artificial artifact targets and improve the quality of targets imaging. In [8], for the problem of SAR imaging in interrupted mode, an interpolation method is proposed to complete the missing data. However, the interpolation method requires a large number of continuous data blocks. When the amount of missing data is small, a small deviation can be guaranteed. When the amount of missing data is large and the data are periodically continuously missing, the interpolation methods cannot be used to complete the missing data. Modern spectral analysis methods can predict and extrapolate time series, and it can be used to improve the quality of SAR missing data imaging and eliminate ghost targets. In [9], an approximate maximum likelihood estimator for missing data autoregressive (AR) model is proposed. The algorithm based on AR model relies heavily on the model parameters. For the SAR echo signal with missing data, it is usually difficult to determine the accurate model parameters, so the quality of reconstruction image is very poor.

Due to the serious deficiencies in the SAR missing data imaging algorithm based on parametric model, the algorithms based on non-parametric adaptive filter are proposed to solve the problem of SAR missing data imaging. This kind of non-parametric estimate algorithm is mainly divided into two kinds: Gapped-data Amplitude and Phase Estimation (GAPES) algorithm and Missing data iterative adaptive algorithm (MIAA) algorithm. The GAPES algorithm uses the minimum mean square error criterion to estimate the missing data, which has good robustness to model errors and noise, but has a large calculation burden [10,11,12]. Compared with the GAPES algorithm, the MIAA algorithm has less computational burden, it estimates missing data based on weighted least squares criterion, and it also has superior performance under the condition of large amount of missing data [13,14,15]. In the non-parametric model method, the complex covariance matrix inversion is required. For SAR echo data, some covariance matrices are singular, and there is no inverse matrix, so the calculation error is relatively large. In addition, the SAR echo signal is not strictly added by single-frequency signals, there are motion errors, range cell migration and noise in the SAR phase history data. Using the non-parametric spectral estimation to reconstruct the missing SAR data could not effectively solve the problems of targets ambiguity and artificial artifact targets.

In recent years, SAR imaging theory using sparse optimization methods has been extensively studied. When the radar observation targets scene is sparse, the sparse optimization theory can be used to reconstruct the incompletely SAR echo sampling data to obtain the high-quality 2D observation targets image [16,17]. The compressed sensing theory is a breakthrough in sparse optimization methods. The theory shows that under the condition of sparse transform domain, the original signal can be reconstructed from small data which is less than Nyquist sampling rate. The SAR reconstruction imaging method based on compressed sensing theory can obtain well-focused image from not only the complete SAR data but also the missing SAR data. The traditional SAR imaging system acquires data according to Nyquist sampling theorem. When the SAR echo data is missing, the Nyquist sampling theory is no longer satisfied. Therefore, the compressed sensing theory can be used to solve the problem of SAR missing data imaging. In [18], considering the principle of SAR imaging, the theoretical framework of SAR imaging using the accurate observation model is proposed. In the accurate observation model, orthogonal matching pursuit (OMP) algorithm is used to reconstruct targets image, which significantly improves the quality of SAR imaging. In [19], a reconstruction algorithm based on L_1/2_ norm constrained optimization is proposed, which can effectively reconstruct the image with less sampling data. 

However, the compressed sensing SAR imaging using the accurate observation model has a large amount of computational complexity and it is suitable for SAR reconstruction imaging with a small size echo data. In large amount of SAR imaging, segmented reconstruction is generally used [20]. In [21], the sparse SAR imaging framework based on the approximate observation model is proposed. This theoretical framework can directly reconstruct the image for large amount of SAR echo data. It is a sparse SAR imaging theoretical framework that can be applied in practice at present. In [21], the approximate SAR observation model with iterative shrinkage thresholding (IST) algorithm are used to reconstruct targets image with the sparse scene SAR echo data. The computational memory consumption is significantly reduced. Meanwhile, the high-quality reconstructed targets image can be obtained.

Under the condition that the observation targets are sparse, the methods used compressed sensing theory can effectively reconstruct targets image with the SAR echo signal missing data. In the case of complicated observation targets scenes, the method of using compressed sensing theory has serious shortcomings. Aiming at the shortage of periodically missing data SAR imaging by spectral estimation method and compressed sensing method, in this paper, the imaging method of SAR azimuth periodically continuous missing data based on the RELAX algorithm is proposed. When there are periodic gaps in the azimuth of SAR phase history data, it is equivalent to multiplying the complete signal and a periodic rectangular window function in the signal time domain. The RELAX algorithm is a classic spectrum estimation algorithm. It does not make any limiting assumptions about noise and clutter. It estimates the parameters of the sinusoidal signal by nonlinear least-squares criterion, and it has strong adaptability. The RELAX algorithm [22,23,24] is used to separate the complex sinusoidal signal through continuous iteration to get the ideal complete signal.

However, when there is a large amount of data, the calculation process of the RELAX algorithm is very time-consuming. In this paper, SAR echo data is divided into sub-blocks along the azimuth. For each sub-block, the RELAX algorithm is used to recover the missing part, and then each block recovered data is spliced into the data of the whole observation scene. Through the block processing, the computation complexity is greatly reduced. The algorithm proposed in this paper can effectively eliminate the azimuth artifact targets. The point targets simulated SAR echo data processing and real raw SAR echo data processing verify the effectiveness of the proposed algorithm.

The rest of this paper is organized as follows: in Section 2, the model of SAR azimuth periodically missing data is introduced. The principle and the algorithm flowchart based on the RELAX algorithm are described in detail in Section 3. In Section 4, the simulated point targets SAR echo data and real raw SAR echo data are processed, and the superiority of the algorithm proposed in this paper is demonstrated. Conclusions are given in Section 5.

## 2. SAR Azimuth Periodically Missing Data Model

In this part, the problem of SAR azimuth periodic continuous missing data imaging is analyzed in detail, the influence of periodic continuous missing on SAR targets image is explained in detail, and the novel solution method is proposed.

### 2.1. Stripmap SAR Imaging Model

**Figure 1:**
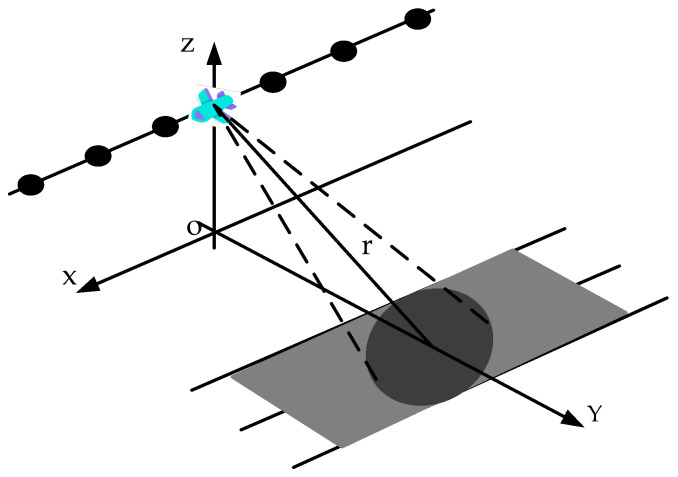
Radar flight geometry.

At present, modern SAR imaging systems generally have multiple operation modes, and the most typical SAR observation mode is the stripmap SAR imaging model. Taking the stripmap SAR imaging mode as an example, the problem caused by the periodically missing data along the azimuth is explained. The simple stripmap mode SAR imaging geometry is shown in Figure 1. In stripmap SAR imaging mode, the antenna beam pointing is fixed. The targets reflection signal received by the radar after demodulation can be expressed as:

(1) $$\begin{array}{cc} s_{0}\left(\tau ,\eta \right) & =A_{0}\omega_{r}\left(\tau -2R\left(\eta \right)/c\right)\omega_{a}\left(\eta -\eta_{c}\right)*\\ & \exp\left\{-j4\pi f_{0}R\left(\eta \right)/c\right\}*\\ & \exp\left\{j\pi K_{r}{\left(\tau -2R\left(\eta \right)/c\right)}^{2}\right\} \end{array}$$

where, *A*_0_ is any complex constant, *c* is the speed of light, *τ* is the range fast time, *f*_0_ is the radar center frequency, *K_r_* is the range chirp frequency modulation rate, *R*(*η*) is the instantaneous slant distance, *ω_r_*(*τ*) is the range envelope, *ω_a_*(*η*) is the azimuth envelope, *η* is the azimuth slow time, *η*_c_ is the beam center deviation time.

**Figure 2:**
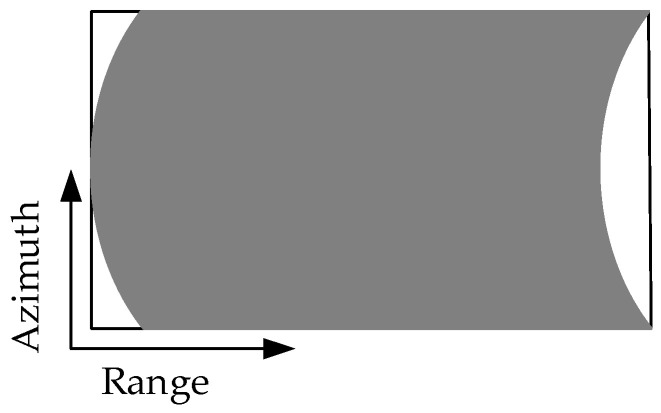
Schematic diagram of two-dimensional (2D) echo with azimuth complete sampling data.

**Figure 3:**
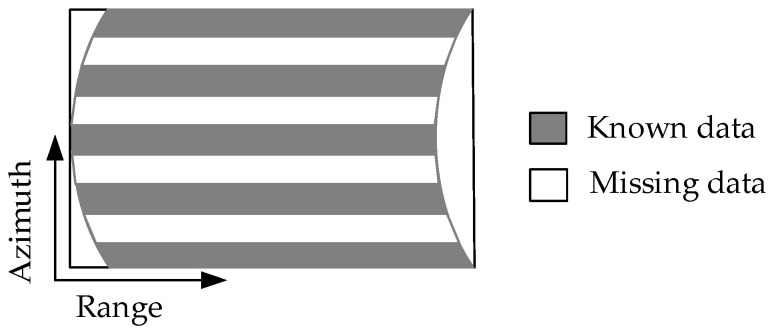
Schematic diagram of 2D echo with azimuth periodic missing data.

In ideal circumstances, when the radar platform flies for a period, the echo signals received by the radar can be recorded in a 2D matrix, as shown in Figure 2. When there is periodic continuous missing data along the azimuth, the 2D echo data matrix is no longer a complete matrix, but periodic notches appear in SAR azimuth, as shown in Figure 3. Using the traditional imaging processing based on Fourier transform, there will be artificial artifact targets and targets energy divergence along the azimuth, which will greatly deteriorate the image quality.

### 2.2. SAR Imaging Processing Algorithm

The rapid development of modern digital signal processing technology provides a solid theoretical basis for SAR imaging processing technology. Since the echo signal received by the radar is divergent signal, further focusing processing is required to obtain a 2D targets image. At present, many kinds of mature SAR signal processing algorithms have been developed [25,26]. In order to solve the problem of artificial artifacts caused by azimuth periodically missing data, a novel imaging processing algorithm is proposed in this paper.

First of all, for the 2D echo data matrix, it is complete sampling data in the range direction, and there are only periodic notches in the azimuth direction. Therefore, the pulse compression technique can be used to compress the SAR 2D echo signal in the range direction. The reference function of range matched filtering is as follows:

(2) $$H_{rc}\left(f_{\tau }\right)=\mathrm{rect}\left\{\frac{f_{\tau }}{\left|K_{\tau }\right|T}\right\}\exp\left\{+j\pi \frac{f_{\tau }^{2}}{K_{\tau }}\right\}$$

The output of range pulse compression is:

(3) $$\begin{array}{cc} s_{rc}\left(\tau ,\eta \right) & =\mathrm{IFFT}\left\{S_{0}\left(f_{\tau },\eta \right)H_{rc}\left(f_{\tau }\right)\right\}=\\ & A_{0}p_{r}\left[\tau -2R\left(\eta \right)/c\right]\omega_{a}\left(\eta -\eta_{c}\right)*\\ & \exp\left\{-j4\pi f_{0}R\left(\eta \right)/c\right\} \end{array}$$

where, *p_r_* (*τ*) is the range envelope after compression, generally is in the form of sinc function. After the range pulse compression, it is necessary to interpolate to correct the range cell migration. The signal after range cell migration correction can be expressed as:

(4) $$\begin{array}{cc} S_{2}\left(\tau ,f_{\eta }\right) & =A_{0}p_{r}\left[\tau -\frac{2R_{0}}{c}\right]W_{a}\left(f_{\eta }-f_{\eta_{c}}\right)*\\ & \exp\left\{-j\frac{4\pi f_{0}R_{0}}{c}\right\}\exp\left\{j\pi \frac{f_{\eta }^{2}}{K_{a}}\right\} \end{array}$$

The azimuth pulse compression operation is performed after range cell migration correction. The reference function of azimuth compression is:

(5) $$H_{az}\left(f_{\eta }\right)=\exp\left\{-j\pi \frac{f_{\eta }^{2}}{K_{a}}\right\}$$

where, *K_a_* is the azimuth frequency modulation (FM) rate. The output after azimuth compression is:

(6) $$\begin{array}{cc} s_{ac}\left(\tau ,\eta \right) & =\mathrm{IFFT}\left(S_{2}\left(\tau ,f_{\eta }\right)H_{az}\left(f_{\eta }\right)\right)=\\ & A_{0}p_{r}\left(\tau -\frac{2R_{0}}{c}\right)p_{a}\left(\eta \right)\exp\left\{-j\frac{4\pi f_{0}R_{0}}{c}\right\}*\\ & \exp\left\{j2\pi f_{\eta_{c}}\eta \right\} \end{array}$$

where, *p_a_*(*η*) is the azimuth envelope after compression, *R*_0_ is the shortest distance to the scene center, and $f_{\eta_{c}}$ is the Doppler center frequency.

**Figure 4:**
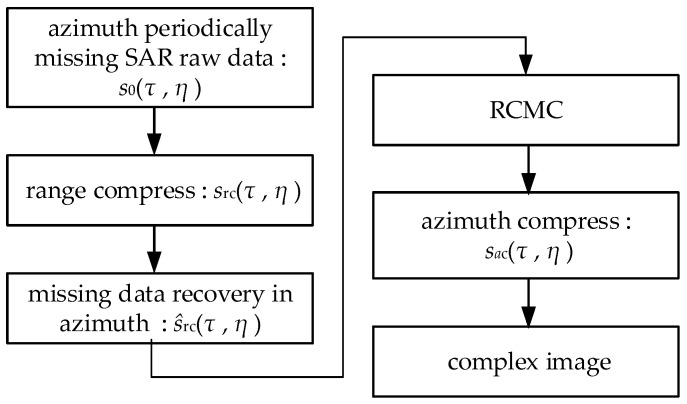
Flowchart of the proposed imaging method.

After the above processing of SAR echo data, high resolution 2D images will be obtained. However, when there are periodic notches along azimuth direction, after the range compression, range cell migration correction and azimuth compression cannot be performed. It is necessary to recover the missing data and get the complete echo data for imaging processing. Therefore, the flow diagram of the algorithm proposed in this paper is shown in Figure 4.

The key step of the algorithm proposed in this paper is how to effectively estimate the missing data. Based on the classical RELAX algorithm, the missing data restoration method is proposed in this paper. This method can effectively reconstruct the missing data and realize the focus imaging processing with the periodically missing data along the azimuth.

## 3. Periodically Missing Data Reconstruct Based on the RELAX Algorithm

At present, a variety of missing data recovery algorithms have been proposed. Among them, the model-based reconstruction method is difficult to estimate the model parameters accurately. The sparse optimization-based method can effectively reconstruct only the sparse targets, the validity of reconstruction echo data of complex observation targets is very poor. The algorithm proposed in this paper can effectively recover the echo data of complex observation targets, and get high quality SAR focused image. The specific algorithm processing steps are as follows:

### 3.1. Azimuth Preprocessing—Reference Phase Multiplication

The time domain signal *s_rc_*(*τ*, *η*) after range pulse compression is multiplied by the azimuth reference phase function to eliminate the secondary phase in the azimuth FM signal. It is convenient to deal with the RELAX algorithm. The specific azimuth reference phase is:

(7) $$h_{ref}\left(\eta \right)=\exp\left(j\pi K_{a}\eta^{2}\right)$$

After multiplying the reference phase, the signal in the azimuth direction only retains the linear phase, and the specific output signal is:

(8) $$\begin{array}{cc} s_{gap}\left(\tau ,\eta \right) & =s_{rc}\left(\tau ,\eta \right)h_{ref}\left(\eta \right)\\ & =A_{0}p_{r}\left[\tau -2R\left(\eta \right)/c\right]\omega_{a}\left(\eta -\eta_{c}\right)*\\ & \exp\left\{-j4\pi f_{0}R\left(\eta \right)/c\right\}\exp\left(j\pi K_{a}\eta^{2}\right)\\ & \approx A_{0}p_{r}\left[\tau -2R\left(\eta \right)/c\right]\omega_{a}\left(\eta -\eta_{c}\right)*\\ & \exp\left\{-j4\pi f_{0}R_{0}/c\right\} \end{array}$$

where, the *R* (*η*) in Equation (8) is approximated.

(9) $$R\left(\eta \right)=\sqrt{R_{0}+V_{r}^{2}\eta^{2}}\approx R_{0}+\frac{V_{r}^{2}\eta^{2}}{2R_{0}}$$

After that, the missing data restoration operation is performed on the obtained output signal.

### 3.2. Missing Data Recovery Based on the RELAX Algorithm

The RELAX algorithm is a high-resolution spectrum estimation method based on the nonlinear least-squares criterion [27,28]. The algorithm does not have any restrictive assumptions for noise and clutter, and it obtains the estimated values of the frequency and amplitude of the signal by nonlinear least-squares criterion. Even under the background of colored noise, this method can obtain the asymptotically effective estimation of each parameter and it has good robustness. The RELAX algorithm has important applications in the field of radar imaging, such as target scattering center extraction, target characteristic extraction and so on.

The SAR echo data after preprocessed in the azimuth direction can be regarded as the superposition of a series of complex sinusoidal signals. The ideal superposition of complex sinusoidal signals can be expressed as the following model:

(10) $$y_{n}=\sum_{k=1}^{K}\alpha_{k}\exp\left(j2\pi f_{k}n\right)+e_{n}$$

where, *n* = 0, 1, 2, …, *N* − 1, *N* is the total number of sampling points, *f_k_* is the signal frequency, *α_k_* is the signal amplitude, and *e_n_* is noise. Define the following vector:

(11) $$\left\{ \begin{array}{l} y={\left[y_{0},y_{1},\cdots ,y_{N-1}\right]}^{T}\\ \omega \left(f_{k}\right)={\left[1,e^{j2\pi f_{k}1},\cdots ,e^{j2\pi f_{k}\left(N-1\right)}\right]}^{T}\\ \Omega =\left[\omega \left(f_{1}\right),\omega \left(f_{2}\right),\cdots ,\omega \left(f_{K}\right)\right]\\ \alpha ={\left[\alpha_{1},\alpha_{2},\cdots ,\alpha_{K}\right]}^{T}\\ e={\left[e_{0},e_{1},\cdots ,e_{N-1}\right]}^{T} \end{array}\right.$$

Then, the model of Equation (10) is written in the form of vector:

(12) y=Ωα+e

Let ***W*** be the periodic rectangular window function, for the SAR echo signal with periodic notches in the azimuth, it can be expressed as:

(13) y=WΩα+e

Let Φ=WΩ, then the Equation (13) can be expressed as:

(14) y=Φα+e

Therefore, the parameter estimation problem of SAR echo signals with periodic notches data in the azimuth is transformed into extracting the frequency and amplitude parameters of the signal according to the model of Equation (14), namely:

(15) $$\left\{{\hat{f}}_{k},{\hat{\alpha }}_{k}\right\}=\underset{\left\{f_{k},\alpha_{k}\right\}}{\mathrm{argmin}}{\left\|y-\Phi \alpha \right\|}^{2}$$

For the optimization problem shown in Equation (15), the RELAX method can be used to extract the frequency and amplitude information.

Assuming that the ${\hat{f}}_{k}$ has been estimated, the ${\hat{\alpha }}_{k}$ can be calculated by the least square (LS) method:

(16) $$\alpha ={\left(\Phi^{H}\Phi \right)}^{-1}\Phi^{H}y$$

Equation (16) is substituted into Equation (15) to get:

(17) $$\left\{{\hat{f}}_{k}\right\}=\underset{\left\{f_{k}\right\}}{\mathrm{argmin}}{\left\|y-\Phi {\left(\Phi^{H}\Phi \right)}^{-1}\Phi^{H}y\right\|}^{2}$$

The ${\hat{f}}_{k}$ can be solved by Equation (17), and then the ${\hat{\alpha }}_{k}$ can be solved by the least square method.

When solving the parameters $\left\{{\hat{f}}_{k},{\hat{\alpha }}_{k}\right\}$, the RELAX method uses the idea of CLEAN technology. The estimated signal is subtracted from the original signal, and the remaining signal is used to estimate the next set of parameters:

(18) $$y_{k}=y-W\sum_{i=1,i\neq k}^{K}{\hat{\alpha }}_{i}\omega \left({\hat{f}}_{i}\right)$$

Combining Equations (16) and (17), the parameters are estimated as follows:

(19) $${\hat{\alpha }}_{k}={\frac{\Phi^{H}y}{\Phi^{H}\Phi }|}_{f_{k}={\hat{f}}_{k}}=\frac{\omega^{H}\left({\hat{f}}_{k}\right)y_{k}}{N}$$

(20) $${\hat{f}}_{k}=\underset{f_{k}}{\mathrm{argmax}}{\left|\omega^{H}\left(f_{k}\right)y_{k}\right|}^{2}$$

The *f_k_* can be estimated from ***y****_k_* by Fourier transform, and the ***α****_k_* can be calculated by substituting *f_k_* into Equation (19). Therefore, the specific steps of using the RELAX algorithm to recover azimuth periodic notches data are as follows:

(1) For the range compressed data after multiplying the reference phase, the first-dimension signal is taken along the azimuth direction, the iteration number *L* is set, and the $\left\{f_{1},\alpha_{1}\right\}$ is estimated by Equations (19) and (20).
(2) Calculate ***y****_k_* according to Equation (18), and use Equations (19) and (20) to estimate the parameters $\left\{f_{2},\alpha_{2}\right\}$. Using the idea of CLEAN technology, repeated iterative calculations sequentially obtain: $\left\{f_{3},\alpha_{3}\right\}$, …, $\left\{f_{L},\alpha_{L}\right\}$, and then use Equation (10) to calculate the complete signal.
(3) Take the second two-dimensional signal along the azimuth direction and repeat steps (1) and (2) to obtain the complete signal of the second dimension. Repeat steps (1)–(3) until the entire two-dimensional matrix is restored.
(4) Multiply the estimated complete two-dimensional matrix signal by the conjugate of the azimuth reference phase function to obtain the estimated complete range pulse compression signal.

**Figure 5:**
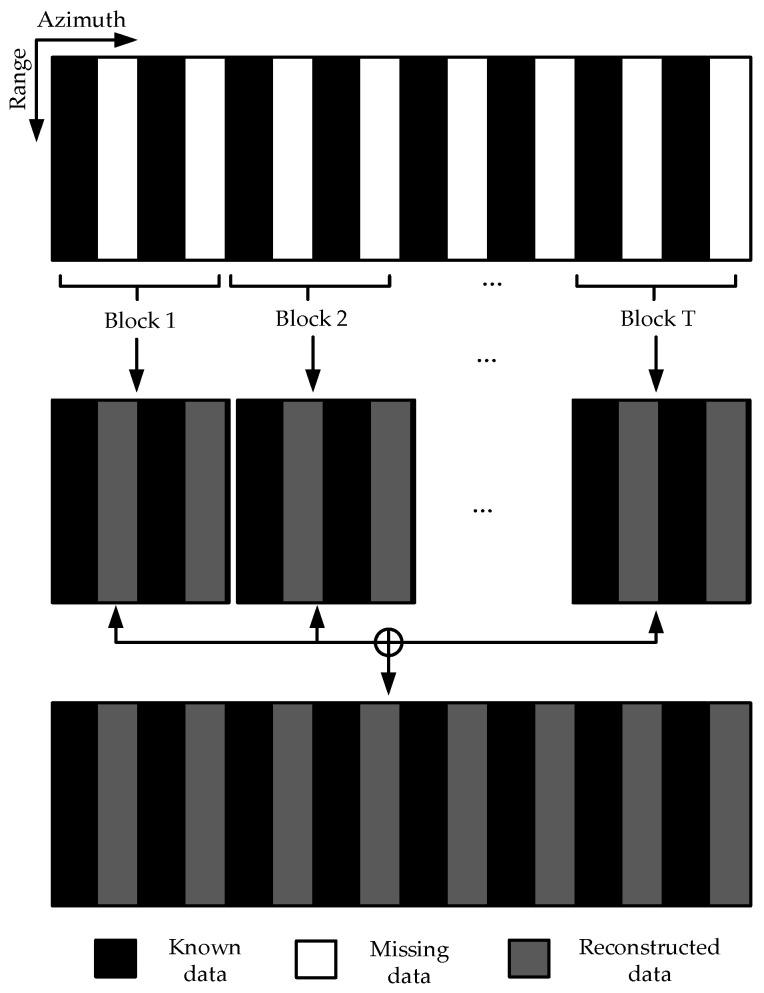
The schematic diagram of 2D echo matrix block restoration.

In the iterative calculation process, the most computationally intensive part is the fast Fourier transform (FFT) operation. Assuming that the size of the SAR 2D echo data matrix is *N* × *M* (azimuth × range), each range gate needs one fast Fourier transforms to estimate a complete single frequency signal along the azimuth direction in each iteration. The complex addition required by the fast Fourier transform is: *N*log_2_*N*, and the complex multiplication is 0.5log_2_*N*, so the calculation amount required to complete each iterative process is: 1.5*N*log_2_*N*. Assuming that each range gate needs to *K* times iterations along the azimuth direction, the calculation amount of the entire iteration process is 1.5*KMN*log_2_*N*. Generally, the SAR 2D echo data matrix has a large scale and the method requires many iterations. Running the algorithm directly on a large-scale matrix requires a huge amount of calculation. Therefore, the method to calculate by dividing into sub-blocks along the azimuth direction is proposed in this paper. The specific blocking strategy is as follows: the matrix is divided into *T* sub-blocks along the azimuth direction in the 2D echo data, each sub-block contains the *N*/*T* points data. The complete data for each sub-block are restored by the RELAX algorithm proposed in this paper, and then each sub-block is spliced into a complete 2D echo data matrix. The schematic diagram of block restoration is shown in Figure 5.

Through block calculation, the number of iterations in each data block is also reduced. Meanwhile, multiple data sub-blocks can be computed in parallel, so the amount of calculation is greatly reduced. Assuming that each data sub-block needs to *k* times iterations, the calculation amount of each data sub-block is (1.5*kMN*/*T*)log_2_(*N*/*T*). Therefore, compared with the algorithm proposed in this paper for the whole 2D echo matrix, the calculation amount can be significantly reduced through block restoration.

In this section, the implementation process of SAR azimuth periodically missing data restoration method based on the RELAX algorithm is introduced in detail, and the block operation is proposed to reduce the amount of computation in the calculation process. Next, the performance of the proposed method is demonstrated by simulation experiments.

## 4. Experiment

In the previous section, the influence of SAR azimuth periodically missing data was analyzed, and the model of periodically missing data was introduced. The missing signal restoration method based on the RELAX algorithm was proposed, and the block restoration was adopted to improve the computational efficiency. The advantages of the proposed algorithm are demonstrated by processing the point-target simulated data and the real raw SAR echo data acquired by Sentinel-1 spaceborne radar. The main parameters of the simulated radar system and the Sentinel-1 radar system are shown in Table 1. All experiments in this paper were performed on a 4-core 2.5 Ghz Intel i5-7300HQ CPU with 16 G memory laptop computer.

### 4.1. Point Target Simulated Data

The simulated echo data of point targets is generated by MATLAB, and the periodic continuous missing data mode is adopted in the SAR 2D echo matrix along the azimuth direction. The specific width of each missing notch is 16 points, and the missing data rate along azimuth direction is 50%. In the simulation, there are three point targets at different positions in the observation scene, and each point target has the same reflection coefficient. The normalized reflection coefficient of the three point targets is set to 1.

**Figure 6:**
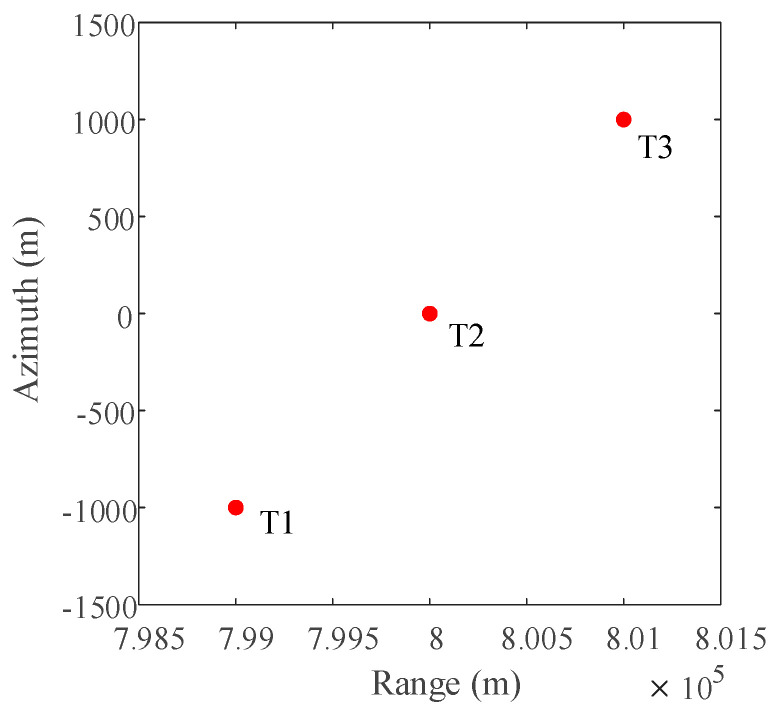
The Distribution of point targets.

**Figure 7:**
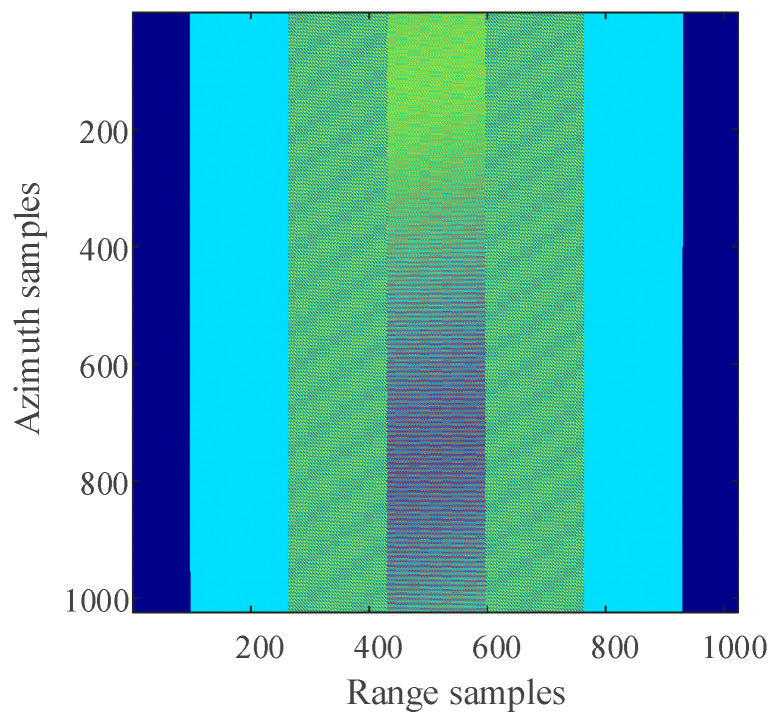
The Schematic diagram of azimuth full sampling echo data.

**Figure 8:**
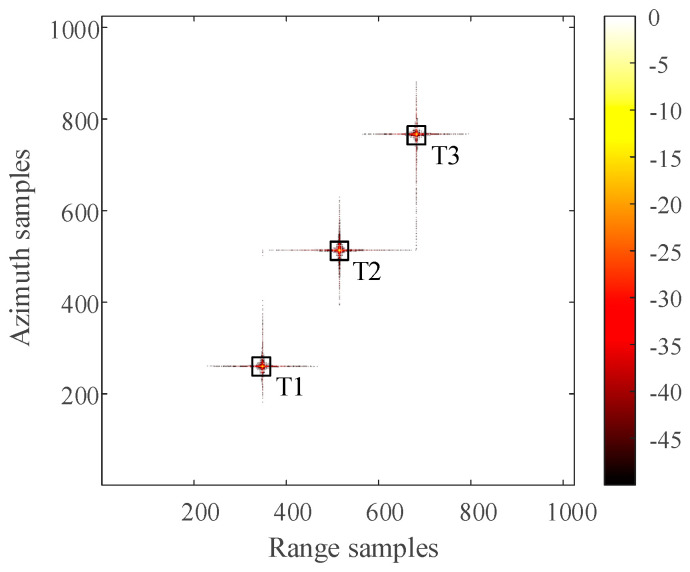
The imaging result of three-point targets with the range-Doppler (RD) algorithm for azimuth full sampling data (dB).

**Figure 9:**
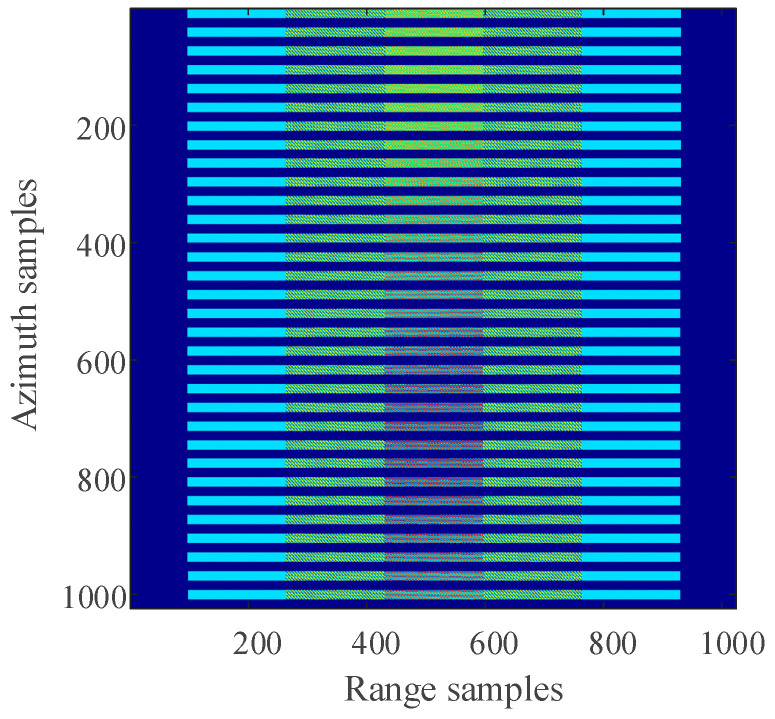
The schematic diagram of periodic continuous missing along Synthetic aperture radar (SAR) azimuth.

**Figure 10:**
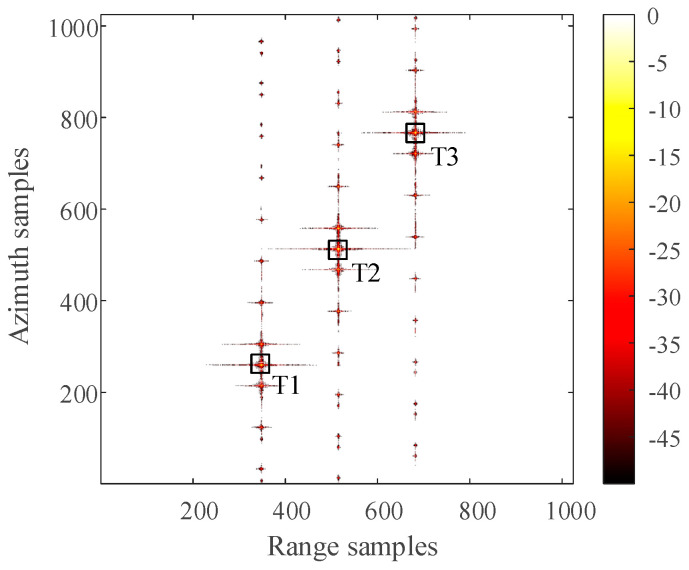
The imaging result of three-point targets with RD algorithm for periodic missing data in azimuth (dB).

The distribution of three-point targets in the observation scene during simulation is shown in Figure 6. The 2D SAR echo data which the size is 1024 × 1024 (azimuth × range) with azimuth full sampling generated by MATLAB is shown in Figure 7. The range-Doppler (RD) algorithm is used to process the azimuth full sampling signal data. The imaging result of the three-point targets is shown in Figure 8. It can be seen that the range-Doppler algorithm can achieve well-focused image with the full sampling echo signal. The schematic diagram of echo data with periodic continuous missing data is shown in Figure 9, where the width of each notch is 16 points. When the missing parts is filled to 0, the range-Doppler algorithm is used for focusing imaging, and the imaging result is shown in Figure 10. It can be seen that for the echo data with periodic continuous missing notches in azimuth, the range-Doppler algorithm is used to focus imaging, there are many artifact targets along the azimuth. The energy of real targets is dispersed, the targets are overlapped along the azimuth, and the imaging quality is greatly reduced.

**Figure 11:**
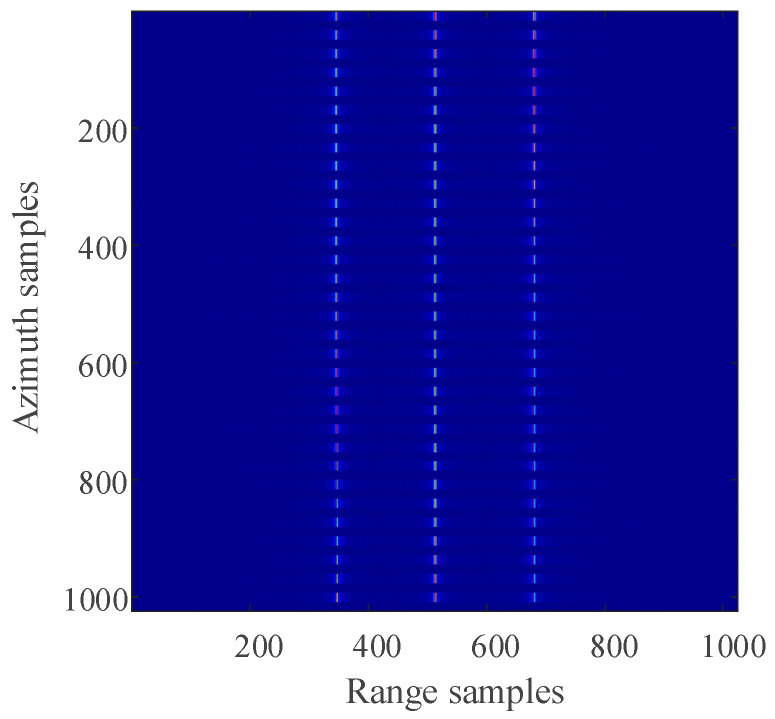
The result of range compression (time domain).

**Figure 12:**
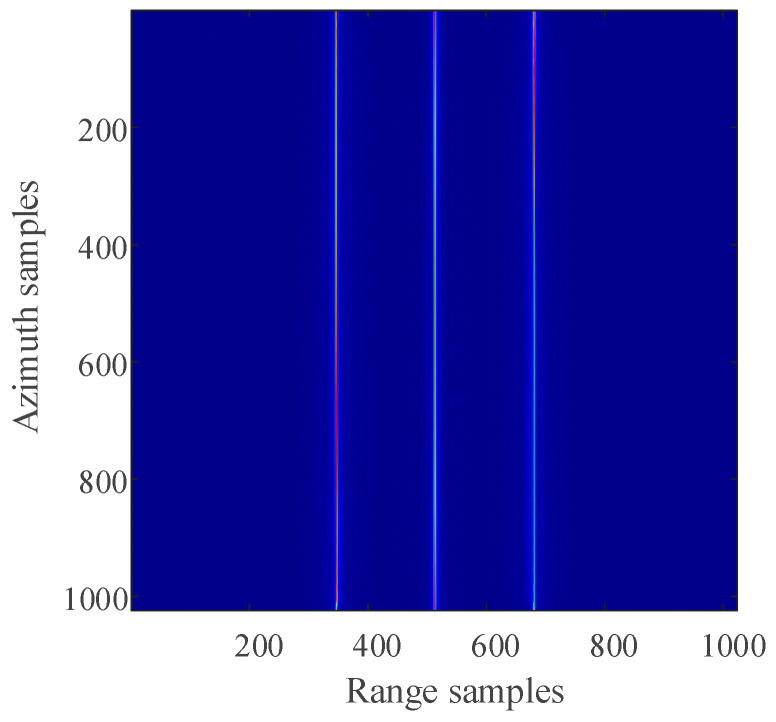
The result of using the algorithm proposed in this paper to restore the missing data (time domain).

**Figure 13:**
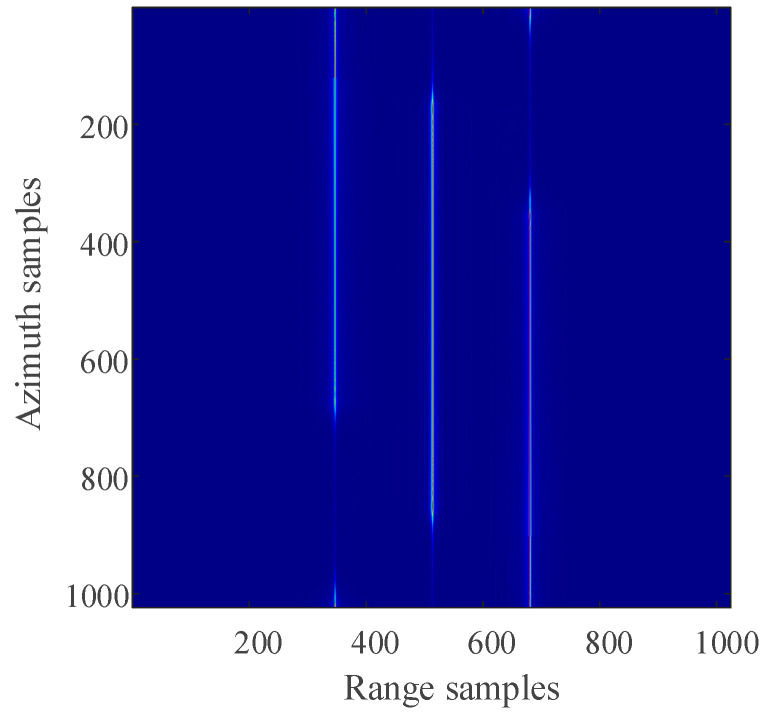
The result of data completion and range cell migration correction (range-Doppler domain).

**Figure 14:**
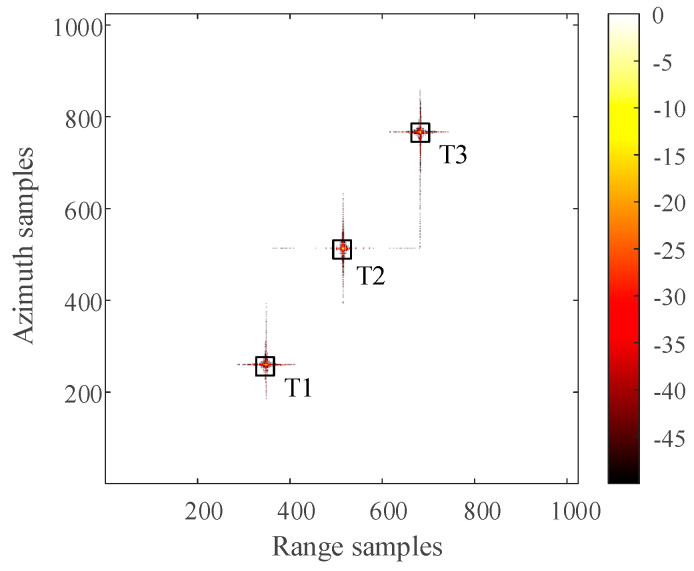
The Imaging result of three-point targets with the algorithm proposed in this paper (dB).

For the 2D SAR echo data with periodically missing notches in the azimuth direction, the algorithm proposed in this paper is used to restore the missing data. First, pulse compression processing is carried out for the complete data along the range direction, and the result of range pulse compression is shown in Figure 11. Because the size of simulated 2D echo data is 1024 × 1024 (azimuth × range), the scale of the 2D matrix is very small. The simulated 2D echo data is not divided into blocks, and the missing data in the whole 2D echo matrix is recovered directly based on the RELAX algorithm. The algorithm proposed in this paper is used to recover the data after range pulse compression, and the result is shown in Figure 12. After the range cell migration correction of the recovered data, the result in the range Doppler domain is shown in Figure 13. The result of azimuth compression is shown in Figure 14. It can be seen from Figure 14 that after processing by the algorithm proposed in this paper, the three-point targets achieve well-focused imaging. The azimuth artificial ghost targets are also significantly suppressed. The points target focused image quality is greatly improved.

**Figure 15:**
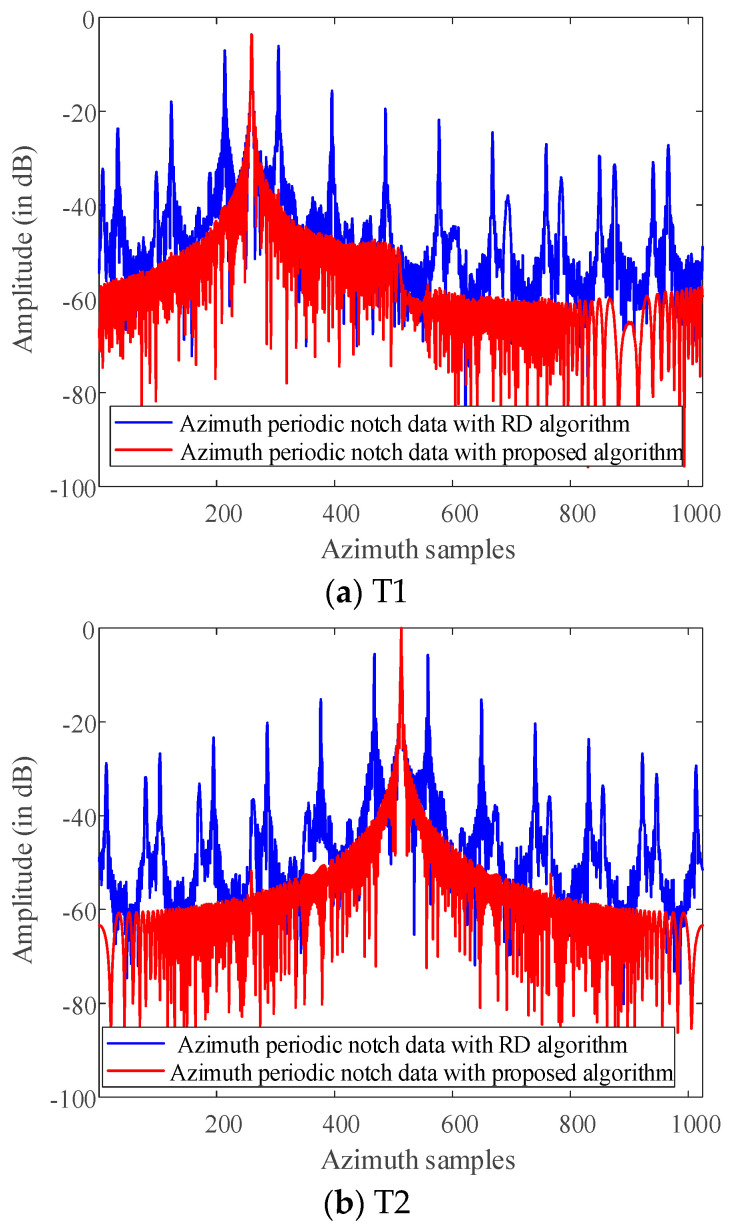

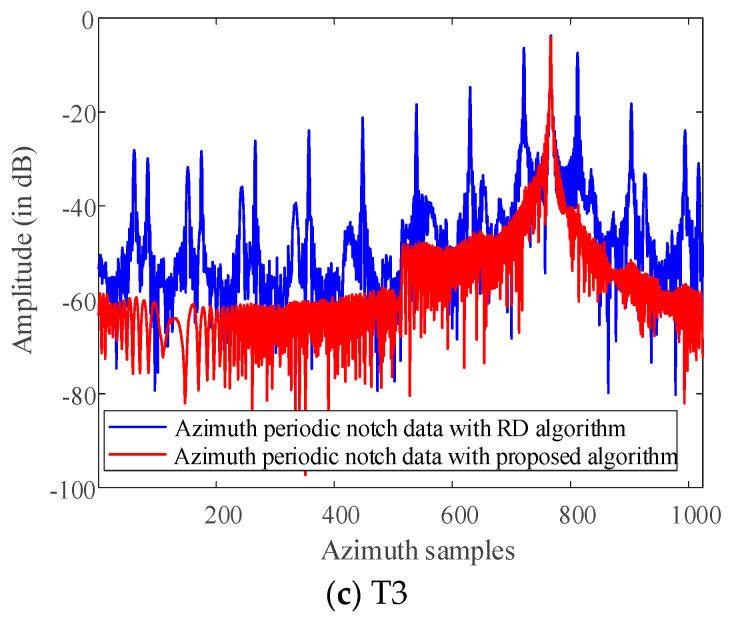
The comparison of slices of three-point targets with different scattering coefficients with RD algorithm and the algorithm proposed in this paper(dB).

The artifact targets suppression of three-point targets in azimuth direction is compared in detail. The range-Doppler algorithm and the algorithm proposed in this paper are used to format image with the periodic notches data along the azimuth direction. The azimuth profiles of the three-point targets are shown in Figure 15. Comparing the slice images of the three-point targets, it can be found that the artificial artifacts caused by the periodic notches data are obviously suppressed, and the real point targets are focused in the azimuth direction.

Generally, when comparing the SAR point targets focusing ability, several important focusing quality parameters such as impact response width (IRW), peak sidelobe ratio (PSLR), integral sidelobe ratio (ISLR) and so on are to evaluate the point targets focus quality. First of all, the three-point targets focus images are obtained by using the RD algorithm with the fully sampled echo data in the azimuth direction, and the focus images are obtained by using the algorithm proposed in this paper with the periodic continuous notches echo data in the azimuth direction. Then, for the focused image, the values of IRW, PSLR and ISLR are calculated along the range and azimuth of the three-point targets, respectively. The detailed data calculated are shown in Table 2. Generally, the ideal focused point target is in the form of SINC function in the range and azimuth, PSLR of SINC function without adding window function is about −13dB, and ISLR is about −10dB.

Analyzing the data in Table 2, it can be seen that the IRW value, PSLR value, ISLR value of three point targets along the range and azimuth direction, for the SAR echo data with periodic notches in the azimuth direction, the result used the algorithm proposed in this paper is very close to the RD algorithm imaging result of the full sampling data in the azimuth direction. Among them, the PSLR value and ISLR value are close to the ideal SINC function value, indicating that the algorithm proposed in this paper can obtain high-quality focused images. Therefore, the algorithm proposed in this paper has better restoration ability for periodic notches data and it can achieve high-performance point targets focus imaging.

Through the point target simulated echo data processing, it is shown that the algorithm proposed in this paper can effectively restoration the SAR 2D echo data with periodic notches in the azimuth. It can realize the focused imaging of the point targets echo signal, obviously suppress the artificial artifact targets caused by the periodic notches, and achieve the very high-performance point targets focusing image. Next, by processing the real SAR observation data collected by sentinel-1 satellite, the advantages of the algorithm proposed in this paper are further proved.

### 4.2. Sentinel-1 Radar Observation Data

**Figure 16:**
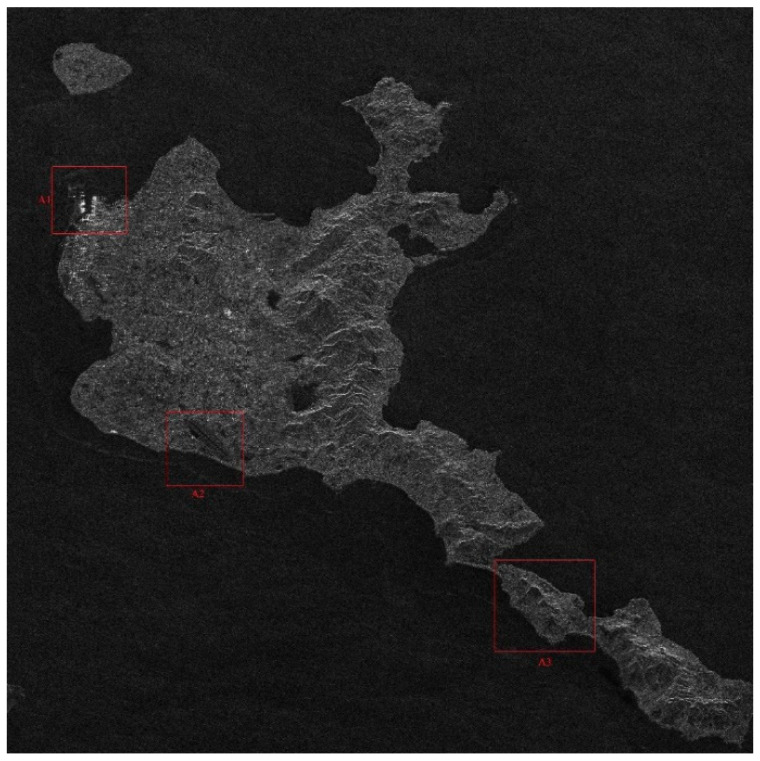
The result of Sentinel-1 radar azimuth full sampling data using RD algorithm (vertical range, horizontal azimuth).

**Figure 17:**
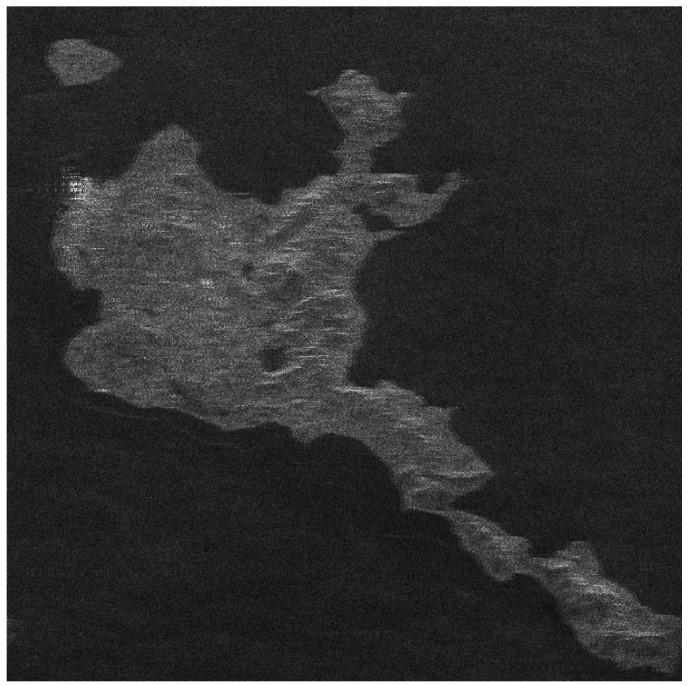
The result of using the RD algorithm when the Sentinel-1 azimuth periodically missing data (vertical range, horizontal azimuth).

**Figure 18:**
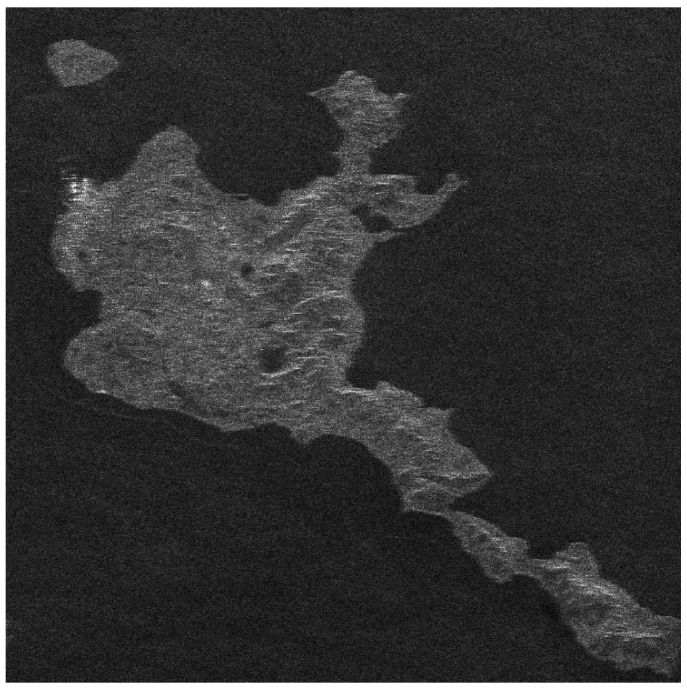
The result of using the BURG extrapolation algorithm when the Sentinel-1 azimuth periodically missing data (vertical range, horizontal azimuth).

There is no airborne or spaceborne synthetic aperture radar operating in azimuth periodic interruption mode. However, considering the principle of periodic continuous missing data, the complete sampling SAR echo data can be periodically removed partial data along the azimuth direction to simulate the echo data with periodic notches. The Sentinel-1 satellite is a C-band SAR satellite launched by European Space Agency (ESA), which can realize all-time and all-weather earth observation, and it has a wide range of applications in the field of remote sensing. The main parameters of Sentinel-1 radar are shown in Table 1. In this section, the raw SAR echo data of the size of 8192 × 8192 (range × azimuth) is selected. The missing form in the azimuth is consistent with the point target simulated data. The 2D echo data is periodically deleted in the azimuth direction, in which the width of each notch is 16 points. To be specific, the Sentinel-1 raw data were converted into missing form by removing 16 rows after every 16 rows (for a notch size of 16 points) in azimuth. For the raw Sentinel-1 radar echo signal with complete sampling data in the azimuth, the result of focusing imaging using the RD algorithm is shown in Figure 16. It can be seen that the RD algorithm can realize focusing imaging of the fully sampled raw echo signal data in azimuth. When the data is periodically missing 50% along the azimuth direction, the missing part is set to 0, and the imaging result using the RD algorithm is shown in Figure 17. Due to the periodic data notches in azimuth, the focused image appears periodic ambiguity targets in azimuth and targets energy dispersion, which seriously reduces the quality of focusing imaging. The BURG extrapolation algorithm is used to predict the missing part, and the imaging result is shown in Figure 18. Although the BURG prediction algorithm can improve the focus image and reduce the artificial ghost targets, there is still blur energy in the focused image. The result of using the BURG extrapolation algorithm is not ideal.

**Figure 19:**
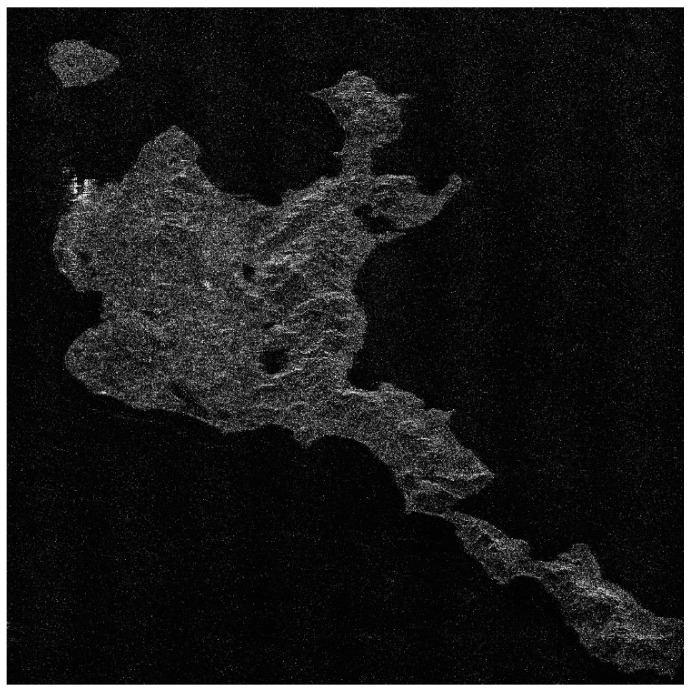
The result of the algorithm proposed in this paper when the Sentinel-1 radar azimuth is periodically missing data (vertical range, horizontal azimuth).

Next, the method proposed in this paper is used to restore and focus the periodically missing data. Because the size of the 2D matrix of the echo data is 8192 × 8192 (range × azimuth) points, and the amount of data is large, the block restoration in azimuth is adopted to reduce the amount of calculation. It is divided into 8 sub-blocks along the azimuth direction, and the matrix size of each sub-block is 8192 × 1024 (range × azimuth) points. Then, the algorithm proposed in this paper is used to recover each sub-block data. Finally, each restoration data sub-block is spliced into the complete 2D data matrix. When the block restoration is not used, the calculation time is about 3286 h. After the block calculation is used, the calculation time is about 55.69 h, and the calculation time consumption is significantly reduced. The imaging result of the algorithm proposed in this paper is shown in Figure 19. Compared with the results of RD algorithm and BURG extrapolation algorithm, it can be found in the result of the method proposed in this paper that the azimuth artifact targets are obviously weakened, the terrain in the obvious area is also effectively imaged, and the targets energy ambiguity is also obviously suppressed. It can be seen that the algorithm proposed in this paper can effectively recover the SAR original phase history data which is periodically missing along the azimuth direction. The well-focused image can be obtained by using the algorithm proposed in this paper.

**Figure 20:**
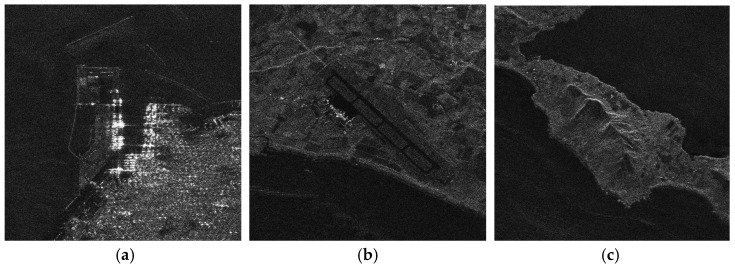
The enlarged view of the three areas in the RD algorithm result when the azimuth fully sampled data. (**a**) Area A1. (**b**) Area A2. (**c**) Area A3.

**Figure 21:**
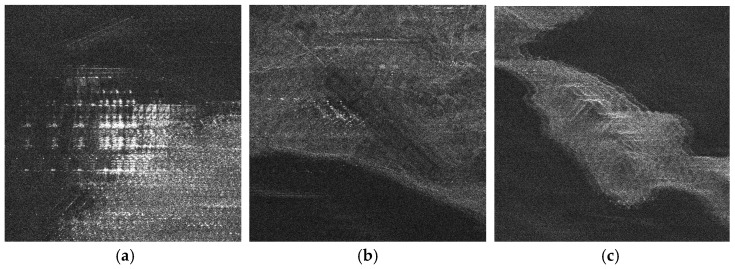
The enlarged view of three areas in the result of RD algorithm when data is periodically missing along azimuth direction. (**a**) Area A1. (**b**) Area A2. (**c**) Area A3.

**Figure 22:**
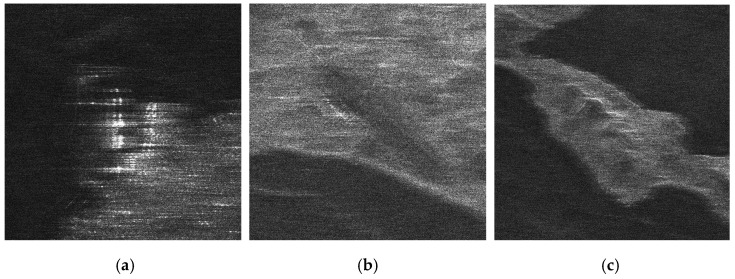
The enlarged view of three areas in the result of BURG algorithm when data is periodically missing along azimuth direction. (**a**) Area A1. (**b**) Area A2. (**c**) Area A3.

**Figure 23:**
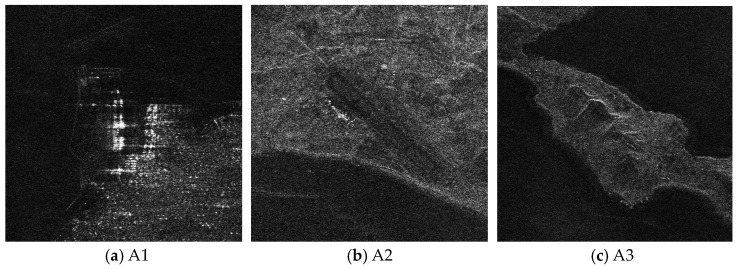
The enlarged view of three areas in the algorithm result presented in this paper when data is periodically missing along the azimuth direction. (**a**) Area A1. **(b**) Area A2. (**c**) Area A3.

The enlarged views of several obvious terrain areas in the entire scene are shown in Figure 20, Figure 21, Figure 22 and Figure 23. Comparing the enlarged images of the three local areas, it can be seen that the algorithm proposed in this paper can effectively suppress strong artificial artifact targets and obtain high-quality focused image. The ambiguity energy of ports, airports and mountains is obviously eliminated. Compared with the focusing result of using RD algorithm directly and the focusing result of using BURG extrapolation algorithm to predict missing data, the focusing image result of the algorithm proposed in this paper is better.

In order to further illustrate the focused imaging performance of the proposed algorithm, the quality of the imaging results of the proposed algorithm is analyzed from several quantitative indexes. In this paper, entropy of image (EoI) and the Structural Similarity Index (SSIM) are used to evaluate the quality of focused images. Generally, image entropy is a statistical measure index that can be used to describe the image texture information [29,30]. For the ambiguity image, the image entropy value is larger, and on the contrary, the well-focused image has smaller image entropy value. SSIM is used to evaluate the similarity of two images. Generally, the greater the SSIM is, the more similar the two images are. The higher SSIM value indicates that the quality of reconstructed image is higher [31].

For the SAR phase history data, the image obtained by the algorithm focus processing is complex image. The entropy of the corresponding complex image is calculated as follows:

(21) $$\mathrm{EoI}=-\sum_{m=1}^{N_{a}}\sum_{n=1}^{N_{r}}g\left(m,n\right)\ln g\left(m,n\right)$$

where, g(m,n) is the scattering intensity density of the image, which is defined as:

(22) $$g\left(m,n\right)=\frac{{\left|S\left(m,n\right)\right|}^{2}}{\sum_{m=1}^{N_{a}}\sum_{n=1}^{N_{r}}{\left|S\left(m,n\right)\right|}^{2}}$$

where, S(m,n) is the scattering intensity of each point in the image, *N_a_* and *N_r_* represent the number of sampling points in azimuth and range respectively. For an image, the smaller the image entropy, the better the focusing effect and the clearer the image.

For two images *x* and *y*, the calculation method of Structural Similarity Index is as follows:

(23) $$\mathrm{SSIM}\left(x,y\right)=\frac{\left(2\mu_{x}\mu_{y}+c_{1}\right)\left(2\sigma_{xy}+c_{2}\right)}{\left(\mu_{x}^{2}+\mu_{y}^{2}+c_{1}\right)\left(\sigma_{x}^{2}+\sigma_{y}^{2}+c_{2}\right)}$$

where, *μ_x_* is the average value of image *x*, *μ_y_* is the average value of image *y*, *σ_x_* is the variance of image *x*, *σ_y_* is the variance of image *y* and *σ_xy_* is the covariance of image *x* and image *y*. $c_{1}={\left(k_{1}L\right)}^{2}$ and $c_{1}={\left(k_{2}L\right)}^{2}$ are constants used to maintain stability. *L* is the dynamic range of the image.

In this paper, the focus image of RD algorithm is selected as the reference image. Generally, for the reconstructed SAR image, the larger SSIM is, the more similar it is to the reference image, the higher the quality of image reconstruction.

In order to quantitatively evaluate the focusing imaging performance of the proposed method, the focusing images obtained by using RD algorithm for complete sampling data in the azimuth, the focusing images obtained by using the RD algorithm when the azimuth periodic continuous missing data, and the focusing images obtained by using the proposed algorithm when periodic notches missing in azimuth direction, the image entropy and structural similarity index of the three images are analyzed. The complex image entropy of three local areas and the whole image in the image are compared. The specific complex image entropy values are shown in Table 3. Compared with the values in the Table 3, it can be seen that for the values with periodic notches in azimuth, whether it is the local image areas or the overall image, the entropy value of the image obtained by RD algorithm increases. The target appears ambiguity, and the quality of the focused image decreases. Using the BURG extrapolation algorithm to predict the missing data, the complex image entropy of focusing imaging results is not significantly reduced. The BURG extrapolation algorithm could not effectively improve the quality of focused imaging. After using the algorithm proposed in this paper, the missing data parts are restored, the entropy value of the focused image is reduced, and the focusing quality of the image is improved.

In order to further illustrate the focusing imaging performance of the algorithm proposed in this paper, the RD algorithm, the BURG extrapolation algorithm and the algorithm proposed in this paper are used to process the SAR echo data with periodic notches along the azimuth, and the structural similarity index of the focus image is compared as shown in Table 4. Compared with the value in the Table 4, it can be found that the SSIM of the algorithm proposed in this paper is large in both the local area and the overall area, which indicates that the image obtained by the method proposed in this paper is more similar to the image obtained by the RD algorithm with complete sampling data in azimuth, so the algorithm proposed in this paper can get high-quality focused image.

In this section, the detailed experimental analysis of the method proposed in this paper is carried out. From the point target simulated data processing and the real raw data processing of the Sentinel-1 radar, it can be shown that the method proposed in this paper can effectively eliminate artificial artifact targets and improve the targets focus imaging quality. Therefore, the algorithm proposed in this paper can effectively solve the problem of SAR azimuth periodically missing data imaging.

## 5. Conclusions

In this paper, the problem of SAR azimuth periodically missing data imaging is analyzed, and a novel method for reconstruction imaging of periodically missing data is proposed. In this paper, the artificial artifact targets and targets ambiguity caused by periodically missing data along the azimuth are analyzed at first. Then, the method to reconstruct periodic notches data based on the RELAX algorithm is proposed. When the size of SAR raw phase history data is huge, it can be reconstructed through partitioning into blocks along the azimuth direction to reduce the amount of computation. When the periodically missing data rate along the azimuth reaches 50%, the method proposed in this paper can effectively suppress the artificial artifact targets and improve the quality of focused image. In the SAR echo data processing experiment of point target simulation, several important parameters such as IRW, PSLR, and ISLR of the point target are analyzed and compared. It shows that the high-quality point target focus image can be obtained by using the proposed algorithm. In the Sentinel-1 radar data processing experiment, by comparing the entropy value of the image and the structural similarity index, it proves that the method proposed in this paper can obviously eliminate artificial artifact targets when there are periodic notches along the azimuth in the SAR echo data, and a well-focused image can be obtained.

## Figures and Tables

**Table 1:** Main parameters of radar system.

Parameters	Simulations	Sentinel-1
Slant range of scene center (Km)	800	820
Radar flight speed (m/s)	7100	7200
Bandwidth (MHz)	15	59.44
Sampling frequency (MHz)	25	66.73
Pules duration (μs)	20	44.172
Carrier frequency (GHz)	5.3	5.45
Pulse repetition frequency (Hz)	1800	1925
Beam squint angle (°)	0	0

**Table 2:** Focusing quality parameter analysis of three point targets.

Targets	Methods	IRW (m)		PSLR (dB)		ISLR (dB)	
		Range	Azimuth	Range	Azimuth	Range	Azimuth
T1	RD algorithm with azimuth full-sampling data	8.71	6.55	−12.31	−13.04	−9.12	−9.78
	Proposed algorithm with azimuth periodic notch data	8.72	6.57	−12.29	−13.13	−9.12	−9.88
T2	RD algorithm with azimuth full-sampling data	8.66	4.92	−12.04	−13.04	−8.89	−9.65
	Proposed algorithm with azimuth periodic notch data	8.67	4.92	−12.10	−13.03	−9.32	−9.65
T3	RD algorithm with azimuth full-sampling data	8.72	6.58	−12.32	−13.04	−9.14	−9.78
	Proposed algorithm with azimuth periodic notch data	8.72	6.67	−12.30	−12.85	−9.13	−9.88

**Table 3:** Comparison of image entropy.

Methods	Entropy of Image			
	Area A1	Area A2	Area A3	Overall Image
RD algorithm with azimuth full-sampling data	11.0737	12.8672	13.6877	17.1222
RD algorithm with azimuth periodic notch data	11.6933	12.9766	13.8011	17.2277
BURG algorithm with azimuth periodic notch data	11.3470	12.9639	13.7702	17.1954
Proposed algorithm with azimuth periodic notch data	10.3239	12.5069	13.2669	16.6218

**Table 4:** Comparison of structural similarity index.

Methods	Structural Similarity Index			
	Area A1	Area A2	Area A3	Overall Image
RD algorithm with azimuth periodic notch data	0.8785	0.6682	0.5213	0.9272
BURG algorithm with azimuth periodic notch data	0.9194	0.6856	0.5415	0.9286
Proposed algorithm with azimuth periodic notch data	0.9275	0.7033	0.6227	0.9325

## Data Availability

Data sharing is not applicable to this article.
